# India Field Epidemiology Training Program Response to COVID-19 Pandemic, 2020–2021

**DOI:** 10.3201/eid2813.220563

**Published:** 2022-12

**Authors:** Sujeet Kumar Singh, Tanzin Dikid, Meera Dhuria, Arti Bahl, Ramesh Chandra, Vaisakh Thandayamparambil Pradeep Vaisakh, Surendra Mohan Prajapati, Nishant Nirwan, Lipsy Paul, Manoj Murhekar, Prabhdeep Kaur, Ganeshkumar Parasuraman, Prashant Bhat, Sheila Longkumer, Kevisetuo Anthony Dzeyie, Pankaj Bhatnagar, Nhu Nguyen Tran Minh, Sukarma Tanwar, Rajesh Yadav, Meghna Desai

**Affiliations:** National Centre for Disease Control, Ministry of Health and Family Welfare, Delhi, India (S.K. Singh, T. Dikid, M. Dhuria, A. Bahl, R. Chandra, T.P. Vaisakh, S.M. Prajapati, N. Nirwan, L. Paul);; National Institute of Epidemiology, Indian Council for Medical Research, Tamil Nadu, India (M. Murhekar, P. Kaur, G. Parasuraman, P. Bhat);; World Health Organization Country Office, Delhi (S. Longjumer, K.A. Dzeyie, P. Bhatnagar, N.T. Minh);; US Centers for Disease Control and Prevention India Country Office, New Delhi, India (S. Tanwar, R. Yadav, M. Desai)

**Keywords:** COVID-19, Field Epidemiology Training Program, India, coronavirus disease, SARS-CoV-2, severe acute respiratory syndrome coronavirus 2, viruses, respiratory infections, zoonoses

## Abstract

The India Field Epidemiology Training Program (FETP) has played a critical role in India’s response to the ongoing COVID-19 pandemic. During March 2020–June 2021, a total of 123 FETP officers from across 3 training hubs were deployed in support of India’s efforts to combat COVID-19. FETP officers have successfully mitigated the effect of COVID-19 on persons in India by conducting cluster outbreak investigations, performing surveillance system evaluations, and developing infection prevention and control tools and guidelines. This report discusses the successes of select COVID-19 pandemic response activities undertaken by current India FETP officers and proposes a pathway to augmenting India’s pandemic preparedness and response efforts through expansion of this network and a strengthened frontline public health workforce.

The COVID-19 pandemic has raised concerns globally about continued vulnerability to infectious disease threats. With a population of >1.3 billion spread across 37 states and union territories, 31 international airports, 11 seaports, 7 ground crossings, and 8 bordering countries, India remains susceptible to global health security threats (*1*,*2*). This vulnerability underscores the need for strengthened core public health capabilities across disease surveillance and laboratory systems, public health workforce, and emergency response. In response to the COVID-19 pandemic, the government of India has reinvigorated its pledge to advance public health capacity through enhanced investments in public health institutions across India and plans to substantially expand the public health workforce.

The Field Epidemiology Training Program (FETP) is a globally recognized workforce development program (*3*–*5*). FETP is a 3-tiered program consisting of a 3-month frontline training program, a 9–18-month intermediate program (Applied Epidemiology Program [AEP]), and a more comprehensive 2-year advanced program (Epidemic Intelligence Service [EIS]) (*5*). India has adopted all 3 tiers of the FETP; program governance is held by central and state departments in close collaboration with the US Centers for Disease Control and Prevention (CDC). These trainings are imparted through 3 hubs: National Centre for Disease Control, Government of India (NCDC); Indian Council for Medical Research–National Institute for Epidemiology; and World Health Organization (WHO) Country Office for India (*6*). The Ministry of Health and Family Welfare steers these trainings through NCDC. FETP alumni (FETP officers trained before COVID-19) are distributed across various multinational, national, state, academic, and nongovernmental institutions in India, enabling the broad dissemination of skills and knowledge gained through FETP. In total, 7 alumni are working with the government of India at the national level, 22 at state government level, 22 with WHO, 7 in various academic institutions, 8 in nongovernmental organizations, and 2 with CDC.

During the pandemic, current and alumni FETP officers were consistently deployed to assist local and state authorities in COVID-19 response activities. FETP officers and FETP alumni at the district and state level were involved in cluster investigations, contact tracing, capacity building and training, response management, surveillance strengthening, infection prevention and control training, and supporting guidelines development. This report describes the successes of the FETP program in India from early 2020 through June 2021 and includes select examples of FETP activities and investigations, both completed and in progress, that have informed response efforts across the country and helped to define the future direction of the India FETP.

## Methods

### COVID-19 Epidemiologic Investigations

**Figure 1:**
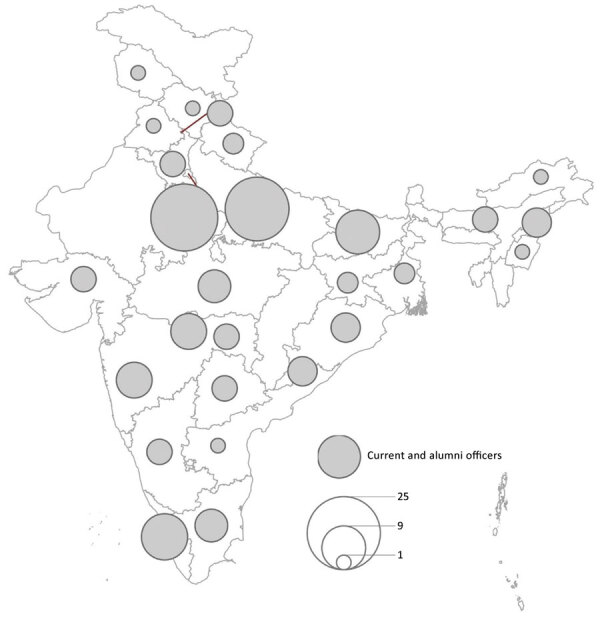
Distribution of India Field Epidemiology Training Program officers (advanced and intermediate current officers and alumni) during COVID-19 response, India, March 2020–June 2021. Circle sizes indicate number of officers.

During March 2020–June 2021, a total of 50 FETP officers (26 officers from AEP and 24 officers from EIS) participated in >44 COVID-19 response projects across India. The 24 EIS officers, 26 AEP officers, and 73 FETP alumni were distributed throughout the country as of June 2021 (Figure 1). EIS officers and FETP alumni were working in 22 (61%) of the 37 states and union territories; the largest concentration of EIS officers and FETP alumni was in New Delhi. As part of the COVID-19 response, broad areas of support from FETP officers to local and state public health authorities were surveillance data analysis and surveillance system strengthening, cluster outbreak investigations in the community and institutional settings such as residential and healthcare centers, epidemiologic studies, and developing and implementing assessment tools for infection mitigation (Table 1). Select FETP officer epidemiologic investigations and their successes are discussed next.

**Table 1:** India Field Epidemiology Training Program (Advanced and Intermediate) projects during COVID-19 response, March 2020–June 2021

COVID-19 response	No. projects
Surveillance system strengthening	11
Surveillance data analysis	5
Capacity building and training	4
Cluster investigation in education institute	4
Community outbreak cluster investigation	3
Epidemiologic study	3
Infection mitigation practices assessment survey	3
Assisted living facility outbreak investigation	2
Case investigation	2
Healthcare facility outbreak investigation	2
Market place outbreak cluster investigation	2
Residential housing complex cluster investigation	2
Assessment of best practices	1
Total	44

Involvement of the India FETP in India’s COVID-19 response began in March 2020 with reporting of initial COVID-19 cases. When India reported its first cluster of COVID-19 in March 2020 in a city in northern India, NCDC deployed EIS officers to develop a containment plan and establish influenza-like illness (ILI) surveillance in the community. Reverse transcription PCR (RT-PCR) testing of potential cases was conducted alongside contact tracing. Using case mapping, 3 containment zones were identified, and >1,700 field teams conducted a door-to-door survey of ILI cases. Over the course of 17 days, 3,561 ILI cases were identified. Of these, 8 cases were laboratory confirmed as COVID-19; of those cases, 3 (38%) case-patients had a history of recent travel outside India, 4 (50%) were in direct contact with a person confirmed to have COVID-19, and 1 (13%) was an indirect contact of a confirmed COVID-19 case-patient (second-generation case). Officers implemented a COVID-19 cluster containment plan that included enhanced surveillance around identified clusters and contact tracing using rapid response teams. The plan was successful in preventing case transmission beyond the second generation.

In March 2020, a COVID-19 cluster was reported among healthcare workers in a cancer hospital in northern India. In the early days of the pandemic, healthcare facilities did not have COVID-19–specific protocols for source reduction. EIS officers led an investigation to identify potential factors associated with these infections and recommended steps to prevent further transmission. Testing and contact tracing identified 25 case-patients, of which 18 (72%) persons were involved in aerosol-generating procedures without following precautions against airborne transmission. Support from FETP officers led to the development of infection prevention and control checklists for healthcare settings and, during the course of the pandemic, served as a successful strategy for ongoing cluster containment activities nationwide. (7).

**Figure 2:**
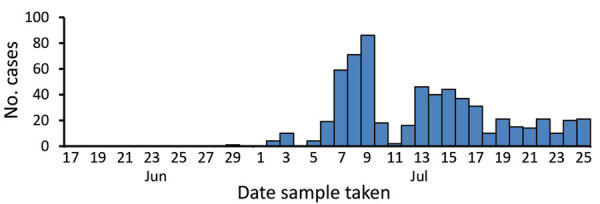
COVID-19 cases in a coastal fishing community in southern India, June–July 2020.

In June 2020, a coastal community in the southern part of India reported 620 COVID-19 cases. An investigation led by an EIS officer was initiated to identify potential risk factors associated with this cluster. A 1:2 case–control study was conducted at the community health center level. A case-patient was defined as a resident of the identified locality who tested positive for COVID-19 during June 17–July 25, 2020. Cases started rising in the first week and peaked in the second week of July (Figure 2). This case–control study demonstrated that 31% (15/49) of case-patients had exposure to the local fish market compared with 10% (9/93) of controls (odds ratio [OR] 3.9, 95% CI 1.4–11), and 37% (18/49) of case-patients had a family member in the fish business compared with 16% (15/93) of controls (OR 2.8, 95% CI 1.2–6.7). These findings underpin the need for adherence to physical distancing to minimize contact with infected persons and the benefit of wearing a face covering or mask.

On March 30, 2021, a COVID-19 outbreak was reported in a residential training institute in the northeastern part of India. Students at the institute came from across India. The infections began after the reopening of training sessions after the lockdown was lifted in February 2021. An AEP officer investigated the outbreak and recommended containment measures. A total of 114 COVID-19–positive case-patients (111 confirmed through RT-PCR) were identified (attack rate 99%). The median patient age was 24 years (range 1–78 years), and 27 (24%) were symptomatic. Of the 114 case-patients, 98 (86%) were students; the remainder were faculty and staff. The investigation led to the closing of the school and implementation of school infection control assessment tool (*8*).

### Operational Support Activities

**Figure 3:**
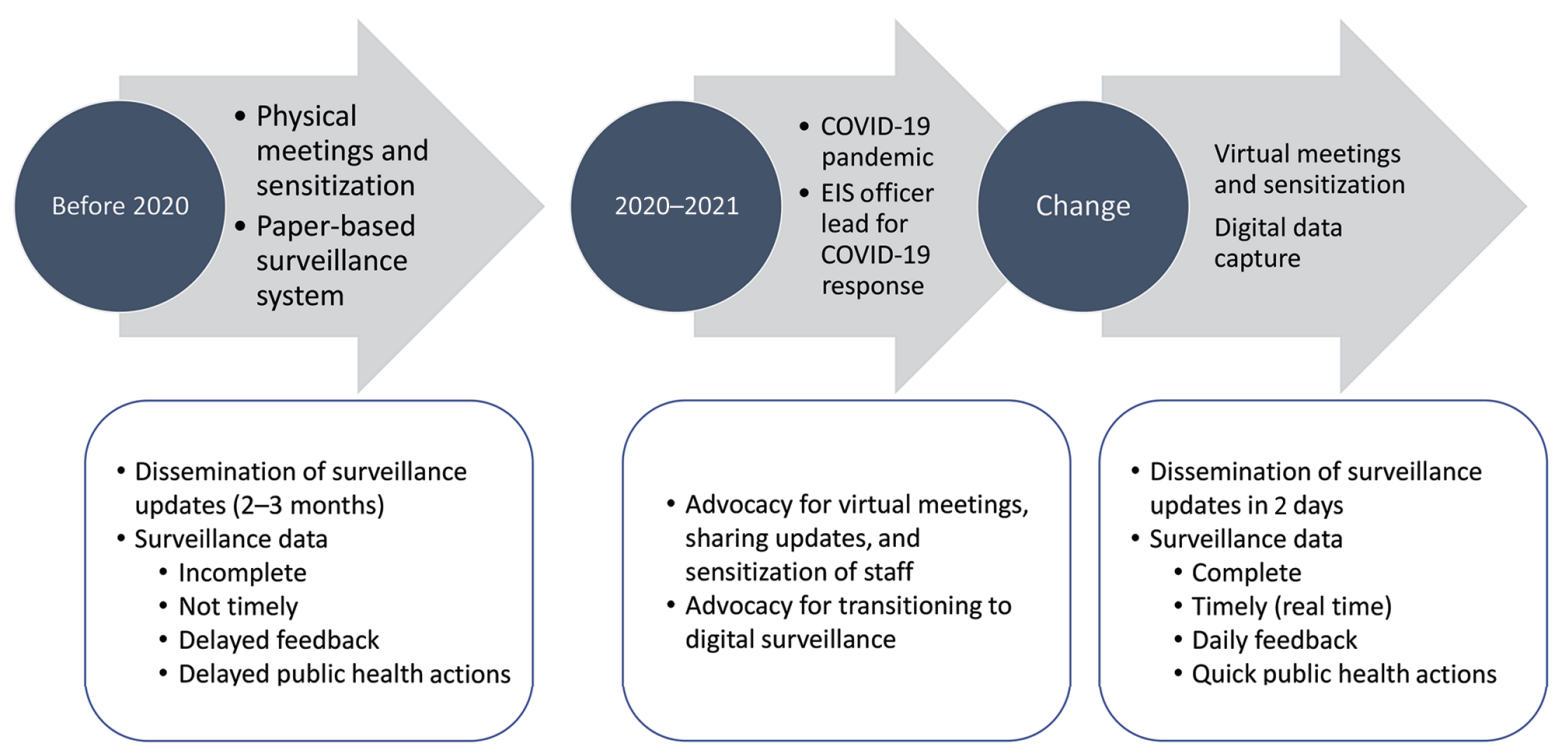
Implementation changes in surveillance activities in Udupi, Karnataka, India, 2020–2021. EIS, Epidemic Intelligence Service.

India FETP officers supported district administrations in strengthening local level surveillance systems, which helped authorities closely monitor COVID-19 activity and the delivery of supplies. An EIS officer was appointed as the nodal officer for COVID-19 containment by the district administration of Udupi, a district in southern India. This officer received the mandate to establish a new surveillance system across the district to guide containment efforts. Early in the pandemic, the officer helped the district transition from paper-based forms to digital surveillance using the EpiCollect5 application (https://five.epicollect.net), a free and easy-to-use mobile data-gathering platform. The application proved especially useful in helping to trace returnees from abroad. The new surveillance system used a virtual platform for trainings, meetings, and discussions. With the help of information gathered through the early transition to digital surveillance, revised guidelines were disseminated and implemented in the district within 2 days (rather than months) of their update. This pace has been maintained throughout the pandemic. The local EIS officer in Udupi in the state of Karnataka played a crucial role in implementing changes in surveillance activities in the district (Figure 3)

During the second wave of COVID-19 in India in April 2021, the district of Udupi observed a 100% increase in cases, which stretched healthcare infrastructure and led to a shortage of consumables such as oxygen and diagnostic supplies. An EIS officer appointed as the nodal officer for COVID-19 containment in the district was assigned to assess oxygen consumption. The officer’s analysis revealed that ≈17% (120/750) of hospitalized patients needed ventilatory or high-flow oxygen support during the peak of the outbreak. Because of patient compliance and ease of use, physicians initially preferred high-flow nasal oxygen cannulas for treatment. These continuously use oxygen at the same flow rate during inspiration and expiration, which results in waste. Unlike high-flow nasal oxygen, ventilators or nonrebreathing masks use oxygen only during inspiration, which has been shown to reduce oxygen consumption by 25%–30%. Along with this intervention, an EIS-led team audited oxygen demand in the district hospitals, which calibrated oxygen administration to maintain blood oxygen saturation of 90%–95%. This exercise reduced oxygen requirement by 8%–10%. Under the officer’s supervision, a detailed oxygen delivery pipeline inspection was conducted in major hospitals to find and correct leakage points, saving another 5% of oxygen.

Kumbh Mela, a mass religious gathering that occurs once every 12 years, brings pilgrims from all parts of India and other countries to Haridwar, Uttarakhand. The Kumbh Mela in 2021 was scheduled to take place within a 12.3 km^2^ area during the months of March and April 2021. More than 10 million pilgrims were expected to participate in this religious gathering, essentially doubling the state population over a period of 1 month. The state government provided guidance to attendees on recommended COVID-19–appropriate behaviors, including mask-wearing. WHO and NCDC EIS and AEP officers participated in public health response activities in addition to regular disease surveillance and preparedness duties. FETP officers assessed mask-wearing compliance among Kumbh Mela attendees in Haridwar (6,200 persons observed in 3 weeks) and provided daily updates on COVID-19–appropriate behavior to the administration. Those updates were then used to plan corrective measures. They also assessed acceptance of the COVID-19 vaccine among Kumbh Mela pilgrims >18 years of age and determined factors associated with vaccine hesitancy. Among the interviewed pilgrims, 44% (140/318) had received >1 dose of COVID-19 vaccine, and among the 56% (178/318) unvaccinated persons, 63.0% (112/178) were not eligible for vaccination (persons <45 years of age were not eligible for vaccination in March–April 2021).

## Results

### Policy and Global Health Implications

Many of the COVID-19 cluster investigations and operational support activities undertaken by India FETP officers had policy implications at the state and national level (Table 2). These implications included standardizing infection prevention and control practices in healthcare settings and schools and establishing robust surveillance systems in select districts (*7*,*8*).

**Table 2:** Policy implications of select COVID-19 response efforts by the India Field Epidemiology Training Program, March 2020–June 2021*

Response type	FETP officers	Location and date	Policy implications
COVID-19 cluster containment in the initial phase of the pandemic	2	Northern India, March 2020	Implementation of a coordinated containment plan served as a template for management of COVID-19 clusters in India.
COVID-19 outbreak investigation in a healthcare facility	2	Northern India, March 2020	Infection prevention control checklist developed for routine healthcare delivery during the pandemic (*7*).
COVID-19 outbreak linked to a fish market—a case-control study	2	Southern India, June 2020	Findings underpinned the need for adherence to physical distancing, masking, and implementation of testing and contact-tracing programs in marketplaces during periods of ongoing community transmission.
COVID-19 cluster investigation in a residential training institute	1	Northeastern India, March 2021	Implementation of school infection control assessment tool (*8*).
Support in establishing digital COVID-19 surveillance system	1	Southern India, May 2020	Surveillance system provided flexibility to accommodate changes in the testing and contact-tracing guidelines, resulting in optimal testing and contact tracing.
Monitoring consumption of oxygen during the second COVID-19 wave	1	Southern India, April 2021	Timely interventions reduced the district oxygen requirement by >43% (1,765–1,004 L per patient per day). Similar models were recommended to be replicated in other districts to optimize the oxygen requirement.
Response to Kumbh Mela	16	Haridwar, Uttarakhand, India, March 2021	Daily feedback from FETP officers led to an overall increase of 21% (62%–83%) in mask use and a 14% (31%–45%) improvement in the correct use of masks among those who were already using a mask.
*FETP, Field Epidemiology Training Program.			

The COVID-19 pandemic has highlighted the contribution of trained field epidemiologists in a public health emergency and recognized them as an integral part of the public health workforce on the frontline. Most currently enrolled and alumni FETP officers supported COVID-19 pandemic response work at their respective duty stations. The remainder mentored current FETP officers in investigative and containment activities. The importance of a strong network of trained field epidemiologists is now well accepted at the highest level in the government. A Parliamentary Standing Committee review report on COVID-19 response has recognized the crucial gap of epidemiologists in India’s public health system (*9*). The committee also observed that, per the Workforce Development Action Package under the Global Health Security Agenda, the country needs to work toward achieving the target of 1 trained field epidemiologist per 200,000 population (*10*,*11*).

Although the pandemic has provided insights into strengthening the public health workforce on the frontline (epidemiologists and surveillance officers), it has also pushed the FETP program to become more innovative in curriculum delivery. Officers from all 3 hubs are spread across the country, but the FETP struggled to provide in-person training opportunities for officers because of travel restrictions, which delayed the completion of some core learning activities. However, because the FETP officers were part of the public health system, they received multiple opportunities both through the FETP and their placement sites to participate in COVID-19 response efforts. At each hub, the FETP adopted innovative hybrid instructional and mentoring methods using online platforms such as Zoom Video Communication (https://zoom.us) for induction trainings and remote mentoring sessions to provide learning opportunities for officers.

In addition to highlighting opportunities to modernize curriculum delivery, the pandemic also raised awareness regarding the inequitable distribution of trained public health workers within India. Such inequities in remote rural areas are difficult to address because current public health services are already constrained. This challenge could be managed through training models with an information technology–based learning management system, hybrid models of training, flexible options for placement, and on-site one-to-one mentoring opportunities. The FETP in India is exploring options to develop system tools to optimize learning and training management.

## Discussion

India needs to strengthen its frontline public health workforce with the same level of commitment it has demonstrated in advancing healthcare and clinical medicine over the years. Public health training programs in India provide strong academic knowledge but offer limited hands-on exposure and applied epidemiology skills (*6*). Focused on-the-job epidemiology training, especially for in-service public health personnel, must be provided. The FETP offers the flexibility to provide training that is directed toward the necessary scope of work.

FETP needs support from the Ministry of Health and Family Welfare in establishing professional recognition (in partnership with universities), career pathways, and incentives for trained FETP officers. Recruiting and using FETP officers in public health leadership positions within the government system can help generate support for FETP training at the district and state levels. As a program, gaps in the FETP implementation must be identified and solutions put in place to ensure quality and rapid scale-up. In addition, a strong FETP alumni network in India and other countries can provide the required mentorship to support FETP expansion and response surge capacity in India and the surrounding region.

During the past decade, the FETP in India has trained >70 officers in advanced epidemiology, and >300 epidemiologists and surveillance officers have been trained under the frontline FETP. However, with a population of 1.4 billion, India requires a much larger public health workforce (≈7,000). Both advanced and intermediate FETP has contributed immensely to national and state-level responses to COVID-19, but rapid expansion of the frontline FETP is needed at the district level. Strengthening its public health workforce capacity through the expansion of all 3 levels of FETP is arguably the most critical element of India’s pandemic preparedness and response efforts.
